# Reversible Carrier Modulation in InP Nanolasers by Ionic Liquid Gating with Low Energy Consumption

**DOI:** 10.1002/advs.202412340

**Published:** 2024-12-16

**Authors:** Chia‐Hung Wu, Chi‐Wen Chen, Hung‐Jung Shen, Hsiang‐Yu Chuang, Hark Hoe Tan, Chennupati Jagadish, Tien‐Chang Lu, Satoshi Ishii, Kuo‐Ping Chen

**Affiliations:** ^1^ College of Photonics National Yang Ming Chiao Tung University 301 Gaofa 3rd Road Tainan 71150 Taiwan; ^2^ International Center for Materials Nanoarchitectonics (MANA) National Institute for Materials Science (NIMS) 1‐1 Namiki Tsukuba Ibaraki 305‐0044 Japan; ^3^ Institute of Photonic System College of Photonics National Yang Ming Chiao Tung University 301 Gaofa 3rd Road Tainan 71150 Taiwan; ^4^ Institute of Photonics Technologies National Tsing Hua University Hsinchu 300 Taiwan; ^5^ ARC Centre of Excellence for Transformative Meta‐Optical Systems Department of Electronic Materials Engineering Research School of Physics The Australian National University Canberra ACT 2600 Australia; ^6^ Department of Photonics College of Electrical and Computer Engineering National Yang Ming Chiao Tung University Hsinchu 30010 Taiwan

**Keywords:** carrier modulation, flexible substrate, InP nanolasers, ionic liquid

## Abstract

Nanoscale light sources are demanded vigorously due to rapid development in photonic integrated circuits (PICs). III‐V semiconductor nanowire (NW) lasers have manifested themselves as indispensable components in this field, associated with their extremely compact footprint and ultra‐high optical gain within the 1D cavity. In this study, the carrier concentrations of indium phosphide (InP) NWs are actively controlled to modify their emissive properties at room temperature. The InP NW lasers can achieve repetitive switching between photoluminescence (PL) and lasing with an extinction ratio of 22‐fold by applying a gate voltage of 3 V using ionic liquid (IL) as a dielectric layer. IL brings forth ultra‐high capacitance due to the nanometer‐wide electric double layer (EDL) between interfaces, mapping out gating efficiency of ≈100‐fold compared to the conventional bottom gate configurations. This IL‐embedded nanolaser device can be a promising platform for the advanced integrated nanophotonic system.

## Graphical abstract

This research presents a significant advancement in nanophotonics by utilizing ionic liquid (IL) as a dielectric layer to modulate carrier concentrations in indium phosphide (InP) nanolasers. The study demonstrates efficient, room‐temperature switching between photoluminescence and lasing with a 22‐fold extinction ratio, offering promising applications for integrated photonic circuits.

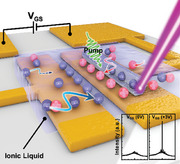

## 1 Introduction

III‐V semiconductor nanowires (NWs) have been studied to a great extent due to their extremely small footprint and capability of enabling strong light‐matter interaction. Thus, promoting themselves to become an indispensable piece in photoelectronic applications, such as memory device,^[^ ^1^ ^]^ photovoltaics, and quantum computing.^[^ ^2^, ^3^, ^4^, ^5^, ^6^, ^7^, ^8^ ^]^ Semiconductor with its high refractive index and in the geometry of a subwavelength wire, offers light to be confined in a 2D guided mode along the wire axis. The end facets of the nanowire provide large optical gain and form the Fabrey‐Perot laser cavity, introducing a nanoscale coherent light source.

To meet the demand for high‐speed data transmission with low energy consumption, two types of approaches have been taken; one is the down‐scaling of integrated circuits to break Moore's Law^[^ ^9^ ^]^ and the other is alternative signal transmission paradigms like quantum communication and photoelectronic chips.^[^ ^10^, ^11^ ^]^ However, past studies on photoelectronic integrated circuits rely on external light sources which are typically fiber‐coupled lasers resulting in size incompatibility with chips becoming even smaller.

Nanowire laser with its extremely small footprint, furnishes nano/micro‐scale waveguiding,^[^ ^12^, ^13^, ^14^, ^15^ ^]^ supports multimode resonance, and provides a coherent light source in the nanoscale.^[^ ^16^, ^17^, ^18^, ^19^, ^20^, ^21^ ^]^ Additionally, carrier dynamics in NWs is a crucial topic as it directly influences the optical and electronic properties of the material. Modulations of NW lasers have been explored by techniques such as chemical doping during the growth process,^[^ ^22^ ^]^ 2D material integrated heterostructures,^[^ ^23^ ^]^ and electro‐doping with gating.^[^ ^24^ ^]^ However, the first two techniques are irreversible after sample fabrication. The gating method like back and top‐gating requires high power consumption (i.e., high gate voltage (V_GS_)) and complex designs to fabricate the insulating layer for NWs modulation. Commonly known oxides for the insulating layer applied in field effect transistors (FETs) such as SiO_2_, Al_2_O_3_, and HfO_2_ typically require high‐temperature fabrication, hindering broad applications such as flexible devices based on plastic substrates. Another solid‐state gating design is the wrap‐gate,^[^ ^25^, ^26^ ^]^ where a thin insulating layer is wrapped around the nanowire using atomic layer deposition (ALD) to prevent leakage. Metal is then wrapped around the nanowire to increase the contact area, significantly enhancing gating efficiency. This configuration is particularly effective for uniaxial nanowire systems, providing a sixfold improvement over plain back‐gate setups for hexagonal nanowires. Solid‐state gating delivers stable and fast electrical switching. However, the high cost of fabrication, the required transparent electrodes like graphene and ITO for optical applications, and the low yield rate highlight the need for alternative approaches.

Ionic liquids (ILs) can manipulate the electrical properties of devices by the organization and accumulation of ions. With ILs, a more energy‐efficient and simplified approach to device modulation can be achieved. A key element steering the functionalities of these devices is through the electric double layer (EDL) formed at the liquid‐solid (target material) interface.^[^ ^27^ ^]^ Previously, Wang et al. have proposed that EDL formation is a two‐step process.^[^ ^28^ ^]^ In the first step, for the liquid‐solid case, molecules in the solution approach collide with the solid surface due to liquid pressure. This forms a strong electron cloud overlap between the solution and the originally neutral solid surface, leading to electron transfer and ion bonding. In the second step, the charged surface of the solid may attract ions of opposite polarity in the solution due to electrostatic force, forming the EDL. To employ this phenomenon in semiconductor devices, an external electric field is applied to emphasize the migration of the ions, actively transferring the designated charges to the target material. For example, when positive bias is applied to the gate electrode, anions in the IL migrate and accumulate at the gate terminal. In contrast, the cations repel from the gate and accumulate around the source terminal, forming EDLs at each interface, and inducing *n*‐type doping of the semiconductor. This induces an ultra‐high capacitance of up to several µF•cm^−2^ across a few nanometer‐thick EDLs. Thus, it is possible to modify the semiconductor's electrical properties with minimal energy consumption.

In this work, a simple three‐step fabrication method was demonstrated to actively modulate carrier concentration at a low voltage in indium phosphide (InP) NW lasers, using IL as the gate dielectric. The modulation was investigated and interpreted through the threshold and wavelength shifts of InP NW lasers’ stimulated emission. Two devices were fabricated and studied in this manuscript; one is a silicon‐based substrate with InP NW on a graphene channel, and the other is a glass substrate with two gold electrodes and InP NW on one of them. Graphene, characterized by its semi‐metallic, flexibility, atomic thickness, and transparent properties, has been verified as an exceptional candidate for the development of wearable or transparent devices. Additionally, the resistivity of graphene is known to be sensitive to carrier concentration change throughout the channel. Leveraging the abovementioned characteristics, graphene has been employed as both a transparent electrode and a monitoring tool to detect variations in ion distribution surrounding the channel in the proposed device. As previously mentioned, doping was induced under gate bias as the IL forms an EDL around the InP NWs at the source terminal. The n‐type doping of InP NW causes a donor band (E_D_) to form in the vicinity of the conduction band (E_C_). At room temperature (≈300 K), these excess electrons in the E_D_ received ≈25.85 meV propelling potential to a higher energy state, eventually filling up the conduction band (CB). A fraction of the excess electrons and carriers excited from the valence band (VB) due to radiation above the bandgap may enter a miniband, inducing population inversion at a lower pumping power leading to a threshold decrease for stimulated emission. As these carriers recombine from a higher energy level state, the radiation loss during recombination exhibited higher photon energy, leading to a blue shift in the lasing peak. The collective occurrence of these phenomena is known as the Moss‐Burstein effect.^[^ ^23^, ^29^ ^]^ Overall, utilizing ILs as gate dielectrics yielded advantages in properties such as high capacitance, low energy consumption, transparency, and compatibility with flexible devices. The proposed concept of integrating IL gating with NW lasers facilitates straightforward modulation and simple fabrication, positioning itself as advantageous for the optoelectronic industry.

## 2 Results and Discussion

### 2.1 Device Fabrication and NW Emissive Properties

**Figure 1:**
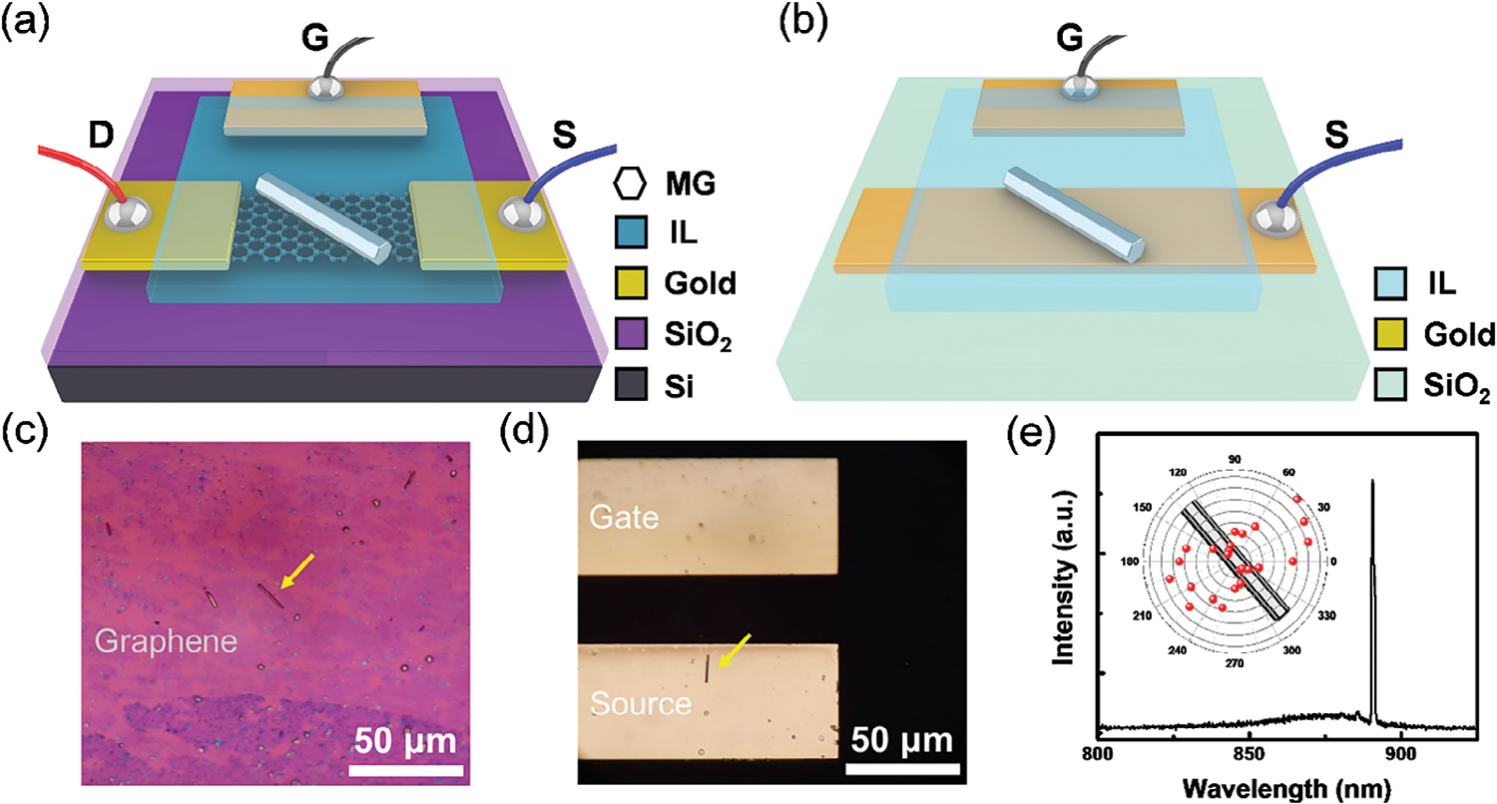
Schematic and emission profile of the device. a,b) Schematic of the IL gate NW setups. Sub‐panel (a) shows a NW on a graphene with a silicon platform (Device A). Sub‐panel (b) shows a universally applicable form of IL‐gated InP NW laser based on a glass substrate (Device B). c) Optical microscope image of NWs on the graphene channel in Device A. The characterized NW was indicated with a yellow arrow. d) Optical microscope image of an InP NW on the source electrode of Device B. e) Stimulated emission spectrum of NW in Device A. Inset: Far‐field emission polarization of the NW.

A schematic diagram of the proposed devices is shown in **Figure** **1**a,b. The silicon photonics‐compatible device is marked as Device A (Figure 1a) and the semi‐transparent device is marked as Device B (Figure 1b) throughout this manuscript. In device A, 300 nm of SiO_2_ was deposited on a silicon substrate. The graphene channel was formed by oxygen plasma etching for 40 s after the chemical vapor deposition (CVD) grown graphene sheet was transferred to the target position,^[^ ^30^ ^]^ which is detailed in Methods. Graphene in the device monitored the gating of the IL and may be seen as an extension of the source electrode, thus simplifying the transfer steps of the InP nanowires. Because of its mechanical stability and interfacial van der Waals force introducing strong adhesion to the substrate, graphene could withstand the polydimethylsiloxane (PDMS) stamping transfer method of InP NWs. The drain, source, and gate electrodes were 100 nm gold deposited by thermal deposition with a shadow mask as depicted in Figure 1a. InP NWs were transferred onto the designated location of the graphene channel by a home‐built transfer system (See Figure S2, Supporting Information). Finally, to form an IL layer, 1 µL of 1‐Ethyl‐3‐methylimidazolium bis(trifluoromethylsulfonyl)imide (EMIM‐TFSI) was drop cast onto the sample and encapsulated the liquid with a cover glass to avoid droplet formation. IL is in contact with the gate and source (graphene) electrodes as shown in Figure 1a. The NW located on the graphene channel was pointed out with an arrow in Figure 1c. Device B was proposed to show the tunable NW laser could be universally applied with just a simple fabrication. As shown in Figure 1b, the gate and source electrodes were 100 nm gold made by a shadow mask and thermal deposition. Because the deposited gold can be ruined during the PDMS stamping process, the InP NWs were transferred to a region adjacent to the source terminal, and then carefully pushed onto the electrode (Figure 1c) with a micro‐manipulator that is typically used for TEM sample pick‐ups. (See Figure S3, Supporting Information for NW location manipulating.) The IL layer was encapsulated with a cover glass to form a gating system. The laser spectral profile of the NW is shown in Figure 1d. According to Figure 1d inset, the emission is polarized normally to the NWs, indicating the excitation of photonic modes.^[^ ^16^, ^31^ ^]^

### 2.2 Characterization and Modal Analysis of InP NWs

**Figure 2:**
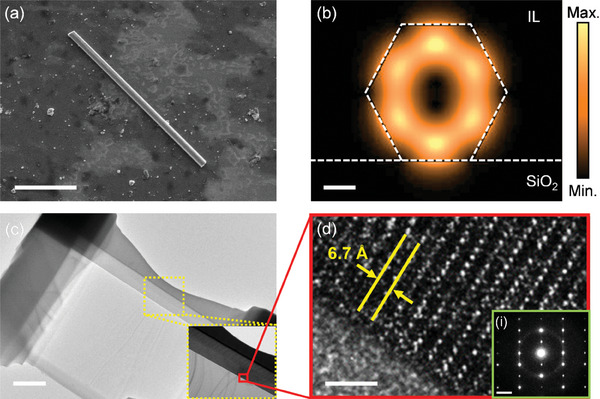
Characterization of the IP NW crystal and mode properties. a) Scanning electron microscope image of the NW on graphene; the scale bar is 5µm. b) Modal analysis of InP NW immersed in IL; the scale bar is 200 nm. c) TEM image of the InP sample; the scale bar is 1 µm. The inset shows a three‐layer structure, silicon, InP, and Pt from bottom to top. d) High‐resolution TEM image of the InP within the red box region of (b), which shows the lattice constant to be ≈6.7 Å; the scale bar is 2 nm. Inset: Selected‐area electron diffraction image of the grown InP; the scale bar is 2 nm^−1^.

The crystal structures of IP NWs are known to be sensitive to growth conditions;^[^ ^32^, ^33^ ^]^ they can be either Zinc Blende (ZB) or Wurtzite (WZ). Emission properties of different crystal formations InP NWs show significant disparity.^[^ ^34^, ^35^, ^36^ ^]^ Thus, it is essential to study the characteristics of the NWs before considering their applications. The SEM image of the NW on graphene is shown in **Figure** **2**a. From the SEM images, the selected NWs for Devices A and B have a length (*l_NW_*) and diameter of ≈13 µm and ≈600 nm, respectively. The modal analysis provides a better understanding of the system supporting rational design. Experimental group index n_g_ of the IL‐gated NW system was extracted from the lasing wavelength. λ_*lasing*_, mode spacing Δλ and cavity length *l_NW_* where $n_{g}=\frac{\lambda_{lasing^{2}}}{2\cdot \Delta \lambda \cdot l_{NW}}$. In Device A, the InP nanowire (NW) is immersed in ionic liquid, the lasing wavelength λ_*lasing*_ is found to be 881 nm, and the mode spacing Δλ is measured at 5.7 nm, as provided in Figure S6b (Supporting Information). From these data, a group index *n_g_* of 5.23 is calculated. Finite Difference Eigenmode Solver (FDE) mode analysis was conducted, with the simulation background refractive index set to 1.42 as the NW is immersed in IL. The computed field profile, as illustrated in Figure 2b, shows that the NW operates in the photonic mode, with substantial field confinement near the InP NW edge. This fits our system design as the electric double‐layer induced carriers are pronounced and limited to the subsurface depth of the NW. For crystallographic analysis, TEM images of the NWs are shown in Figure 2c,d. To prepare TEM samples, platinum was coated on the InP NW for protection and thinned down to ≈50 nm in the axial direction with a focused ion beam (FIB). The selected‐area electron diffraction (SAED) pattern and TEM analysis confirm that the NW was a single crystal, and the structure was WZ as shown in Figure 2d inset. The lattice constant was measured to be ≈6.7 Å, which was in good agreement with the previous studies on WZ InP.^[^ ^37^, ^38^ ^]^

### 2.3 Comparison Between IL and Backside Gated Configurations

**Figure 3:**
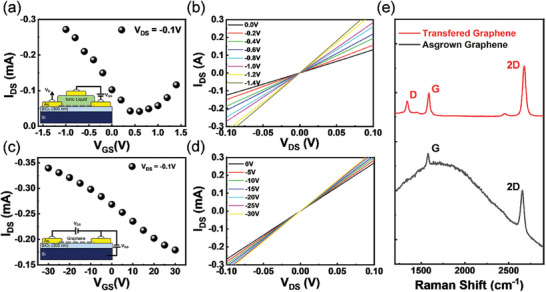
Comparison between IL‐gated and backside‐gated graphene. a,c) Measured *I*_DS_–*V*_GS_ curve under V_DS_ = 0.1 V of IL‐gated and backside‐gated setups respectively. b) The *I*_DS_–*V*_DS_ curves of the IL‐gated setup under V_GS_ = 0 to −1.4 V. d) The *I*_DS_–*V*_DS_ curves of the backside gated setup under *V*_GS_ = 0 to −30 V. e) Raman spectroscopy of the as‐grown and transferred graphene used in Device A. The 2D / G ratio maintained a value ≈2 indicating good quality graphene after the transfer.

In **Figure** **3**, we compare the gating efficiency difference between backside gating and IL gating methods via graphene. It is well known that graphene exhibits the highest channel resistance when the doping level is at the Dirac point.^[^ ^39^, ^40^ ^]^ As a semi‐metal, once the graphene Fermi level is displaced into the conduction or the VB, more free electrical carriers may contribute to the current flow when applied to an external electric field, introducing a lowering of channel resistance. In Figure 3a,c insets show the IL and backside gating method schematics respectively. The IL gating active region was formed by the electric double layer (EDL) introduced by the IL, whereas the active layer in the backside gating configuration is in the deposited SiO_2_. According to the measured results in Figure 3a,c, it is clear that the IL gating setup requires much less gate voltage to modify the graphene Fermi level into the n and p‐type regions. This large contrast in energy consumption originated from the ultra‐high capacitance provided by the IL compared to SiO_2_.^[^ ^41^ ^]^ The remarkable capacitance was due to the formation of EDL in IL that had a thickness of only a couple of nanometers, whereas in the backside gating configuration, the SiO_2_ was 300 nm. Figure 3b,d shows the graphene channel IV curve with respect to different gating voltage levels of IL and backside gating configuration. Additionally, to study the electrical properties of gated graphene, it is inappropriate to ignore the wrinkles and folds that come along with the wet transfer method. The CVD‐grown graphene and its quality were characterized with Raman spectroscopy as shown in Figure 3e. The 2D / G peak ratios before and after transferring graphene both show values exceeding 2, indicating high quality.

### 2.4 Working Principle and Device Performance

**Figure 4:**
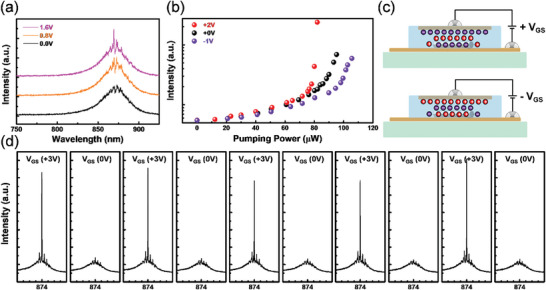
Threshold modulation of Device B under various doping levels. a) The spectrum of NW on Device B under V_GS_ = 0, 0.8, and 1.6 V. b) Light‐in versus light‐out curves of the IL gated NW laser under V_GS_ = −1, 0 and 2V. c) Schematic of carrier dynamics in the system when positive and negative gate biases are applied. At positive (negative) V_GS_, electrons (holes) would accumulate inside the NW cavity, introducing n (p) – type doping. d) Cyclic measurement of NWs under V_GS_ = 0 and 3 V at 78.5 µW pumping energy, showing electrical on and off switching of stimulated emission.

As reported in the literature, the lasing threshold and electrical properties of semiconductor NWs can be tuned according to the doping levels.^[^ ^24^, ^42^ ^]^ However, previous studies regarding threshold modulation in NWs have been mostly focused on field confinement due to nanostructure coupling,^[^ ^21^, ^43^ ^]^ or impurity doping.^[^ ^44^, ^45^ ^]^ Both are not tunable after the fabrication, causing a lack of freedom in tunings. Hereby, the tunability of NWs immersed in IL has been tested by applying different gate voltages to introduce different doping levels. **Figure** **4**a shows an increase in emission power under the same excitation laser power of 78.5 µW. The black line shows the amplified spontaneous emission (ASE) of the NW under 0 V gate voltage, with V_G_ increasing from 0.8 to 1.6 V (orange and purple lines respectively), the stimulated emission (lasing) was clearly observed on the NW, indicating the threshold decrease in the NW lasers. Next, we measured the excitation power‐dependent emission intensity of the NW laser under V_GS_ = −1, 0, and +2 V represented in blue, black, and red colors in Figure 4b, respectively. The mechanism of the observed phenomenon is illustrated in Figure 4c. By applying positive gate voltage, anions (TFSI‐) in the IL aggregate around the gate electrode, while cations (EMIM+) were attracted to the source electrode forming electric double layers (EDLs) at each liquid / solid interface. The NW on the source electrode then be bathed in cations generating an accumulation of negative charges throughout the NW, realizing n‐type doping. The n‐type doping introduced a donor band (E_D_) located in the vicinity of the conduction band (E_C_). At room temperatures (around 300 K), part of the donors contributed as free electrons in the CB. When the above bandgap illumination is introduced, electrons from the VB and donor band are excited to the CB and then eventually fall back down into the VB where carriers recombine, resulting in spontaneous emission. However, some electrons may get trapped in the miniband during the process and as the pumping energy increases, electron accumulation in the miniband forms population inversion, which causes stimulated emission. Note that the n‐type doping from IL decreased the lasing threshold as it introduced excess electrons to the system. This correlates with our experimental results; once the wires are n‐doped (more donors), the threshold of the stimulated emissions drops drastically. On the contrary, if the NW were accumulated by positive charges (p‐type doping), the acceptor band near the VB hinders electrons' transition from VB to CB resulting in an increase in the lasing threshold. To accumulate positive charges in the NW, a negative gate voltage was applied as depicted in the bottom panel of Figure 4c. The robustness of emission functionality of the proposed NW laser concept was tested through a repeated on‐and‐off cycle. Figure 4d shows 5 cycles of V_GS_ = +3 and 0 V, the emission of the NW shows the capability of switching the laser on/off with no obvious degradation. This experiment was tested on different NW lasers with more cycles, and all showed similar trends except for the slight deviations on the V_GS_ for the on‐state. The reason is due to variations in NWs geometry and their intrinsic charged state.

**Figure 5:**
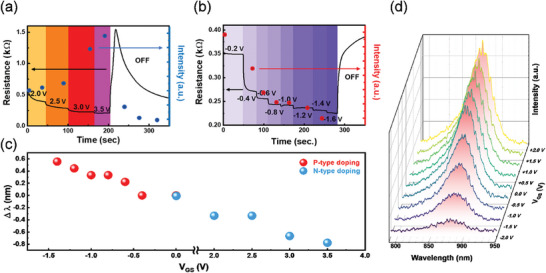
Emissive properties of Device A under various doping levels. Relation between 881 nm stimulated emission intensity and channel resistance of graphene under a) positive and b) negative gate bias. Variations in graphene resistance point out the carrier concentration change throughout the channel. Different color boxes are added for better visibility, referring to different V_GS_ levels. c) Extracted lasing wavelength shift under gate voltage variation, showing a minor blue shift as the NW is gradually n‐doped. The red (blue) data points refer to negative (positive) gate bias assigning p (n) – type doping in the NW. d) PL spectrum of NW on Device A at V_GS_ = −2 to 2 V.

One way to reveal the evidence of NW carrier modulation is to perform Kelvin probe microscopy (KPFM) as it records changes in surface potential. However, it is impossible for surface analysis in this setup because the NWs were immersed in IL and encapsulated under a cover glass. To study charge accumulation, the graphene channel resistance and the lasing intensity at 874 nm of the NW were measured. As shown in Figure 3a, it is clear that the resistance of graphene varies under different doping levels. **Figure** **5**a,b shows the relation between the lasing emission and doping level. As V_GS_ increases (Figure 5a), the Fermi level of graphene moves further into the n‐type regime, causing a decrease in resistance from gate‐induced electrons throughout the channel. The NWs lasing intensity shows a 300% enhancement at V_GS_ = + 3.5 V compared to V_GS_ = 0 V with the excitation power set to 130 µW. Though the enhancement in emission is obvious and shows no trend of saturation, the IL limits the applied gate voltage to ≈4 V due to the electrochemical instability. Note that the pristine graphene used throughout this manuscript is slightly p‐type. We can see in Figure 5a that after V_GS_ = 3.5 V was turned off, the resistance of graphene increased drastically and then dropped gradually indicating the relaxation of gate‐induced carriers throughout the channel (n‐type to neutral to p‐type pristine state). As depicted in Figure 5b, the V_GS_ was given negative voltage introducing anion gathering around the NW, resulting in hole accumulation in the cavity. Opposite to Figure 5a, the NW lasing emission was suppressed and gradually turned off. As the pristine graphene was positively charged, the more negative gate voltage was applied, the smaller channel resistance it exhibited. The lasing peak wavelength (λ_Lasing_) was extracted and shown in Figure 5c. The peak wavelength of NW‐stimulated emission shifts when the charge concentration changes inside the NW cavity. By varying the gate voltage from V_GS_ = −1.6 V to +3.5 V, λ_Lasing_ showed a blueshift of ∆λ = 1.4 nm. Gate‐induced N‐type doping increases the carrier concentration throughout the nanowire cavity, reducing the cavity refractive index as predicted by the Drude model. Whereas P‐type doping decreases the carrier concentration due to the opposite polarity of majority carriers in the as‐prepared intrinsic N‐type InP nanowire. Resulting in the highest refractive index under negative biased conditions and leads to a red shift in emission wavelength. From our observations, the small shift in emission wavelength could also be explained by the Moss‐Burstein effect.^[^ ^29^, ^46^, ^47^ ^]^ When the semiconductor nanowires are subjected to N‐type doping, a high concentration of electrons fills up the energy levels near the bottom of the conduction band (CB). Thus, photo‐excited carriers have to occupy states with a higher energy, resulting in higher photoenergy emission (blue shift) when the excited carriers recombine. In the proposed device, photoluminescence intensity under below‐threshold excitation also varies with different gate voltages, as shown in Figure 5d. The band‐filling model applies to both stimulated and spontaneous emission, as both types are driven by carrier recombination, resulting in similar optical trends, as previously discussed. These optical trends mirror those observed in the lasing and PL experiments, supporting the conclusion that threshold modulation is primarily due to electrical doping induced by the EDL formed by the ionic liquid.

## 3 Conclusion

In summary, this study showcases the concept of dynamically modulating charges in III‐V semiconductor NWs at room temperature by employing ILs as the dielectric gating layer through concise fabrication. Utilization of IL as the dielectric layer enhances the gating efficiency up to 100‐fold compared to the backside gating configuration. This setup only requires a pair of electrodes and is compatible with all insulating platforms, even flexible ones. By applying bias at the gate terminal, anions and cations in the IL aggregated and lined up in a manner with respect to the polarity of the bias, forming EDLs at each solid/liquid interface. The EDL formed around the NW that was placed on the source electrode dopes the NW with electrons/holes, causing the threshold and the lasing peak wavelength to decrease/increase and blueshift/redshift, respectively. The total wavelength shift measured in this manuscript is 1.4 nm, under the gate voltage shift from −1.4 to 3.5 V. In the context of n‐doped InP NWs, a donor band is formed in the vicinity of CB which causes electrons to fill up the lower energy states in the CB. This led to the threshold lowering and blueshift in emission spectrums when optically pumped, as the system contained surplus excited electrons from higher energy states. On the contrary, p‐doped InP NWs exhibited opposite behaviors as the electrons in the system were depleted to a certain level. The proposed setup demonstrated active carrier modulations of III‐V semiconductor NWs, highlighting substantial potential to accommodate the vast applications in the optoelectronic industry.

## 4 Experimental Section

### Growth of InP Nanowires

InP nanowires were grown on a 2 in. (100) InP substrate by using the metal–organic chemical vapor deposition (MOCVD). Temperature and pressure for growth were set to 500 °C and 100 Torr respectively, with a V/III ratio of 80. The grown InP NWs have lengths and diameters ranging from 10 to 20 µm and from 100 to 500 nm, respectively.

### Device Fabrication

Device A was fabricated starting from a 300 nm silicon dioxide deposition by an electron beam evaporation system (ULVAC VT1‐10CE) on a silicon wafer. After the deposition, a graphene sheet was transferred onto the substrate followed by oxygen plasma etching (OMNI‐RIE) for 40 s to form a channel. Gate and source/drain electrodes were thermally deposited 100‐nm thick gold films with a shadow mask. The InP NWs were precisely transferred onto the graphene channel using a PDMS stamp clamped to a probe station as shown in Figure S3 (Supporting Information). Finally, 1 µL of EMIM‐TFSI was drop cast on the graphene channel and encapsulated with a cover glass to avoid the formation of droplets. Device B was fabricated using a glass substrate where the process was compatible with flexible substrates such as PET and PDMS. Gold electrodes of the gate and source were deposited on the substrate. The InP NWs were stamped on the substrate in the vicinity of electrode pads as the PDMS stamp may damage the deposited gold. InP nanowires were pushed carefully onto the source electrode with a micro‐manipulator as depicted in Figure S3b (Supporting Information). Finally, IL was drop cast onto the electrode and encapsulated with a cover glass.

### Graphene Wet Transfer

A chemical vapor deposition (CVD) grown monolayer graphene sheet on copper foil was transferred to the target substrate using the wet transfer method. Prior to the removal of copper foil, graphene was protected by spin coating a thin layer of photoresist (PMMA‐A4) to avoid mechanical damage from fluid tensions during the etching process. The copper foil was etched by floating the stack on Fe(NO_3_)_3_ solution (33 wt%) at room temperature (≈300 K) for ≈12 h. After the etching process, the stack was left with graphene and photoresist. To remove the etchant residue, the stack was cleaned for 1 h using deionized water. The substrate was treated with UV–ozone before the transfer to enhance surface energy, providing a position‐adjustable transfer process without damaging the graphene. The sample was left in a dry cabinet overnight to complete dehydration. After the sample was completely dry, the photoresist was removed by acetone and subsequently washed in isopropanol (IPA).

### Optical Measurements

The InP NW of interest was identified with a visible charge‐coupled device (CCD), then pumped from an incident angle of ≈45 degrees using a 532 nm pulsed laser with a repetition rate of 4 kHz and pulse width of 1 ns. The beam spot was focused with a 5 cm focal length convex lens which resulted in an elliptic shape with short and long axes to be 40 and 130 µm, respectively. The emission from the InP NWs was collected at normal incidence by a 50x objective lens (SLMPLN50X, Olympus) with a numerical aperture of 0.35 and a working distance of 18 mm. The signal was then directed into a spectrometer (Kymera 193i, Andor). To determine the emission polarization as in Figure 1e inset, a rotatable polarizer was placed in front of the spectrometer. Schematic of the above is shown in Figure S4 (Supporting Information).

## Conflict of Interest

The authors declare no conflict of interest.

## Author Contributions

C.‐H.W. performed sample fabrication and characterization. C.‐H.W., T.‐C.L., S.I., and K.‐P.C. analyzed the experimental data. C.‐H. W. and K.‐P.C. wrote the manuscript. All authors discussed the results and commented on the manuscript.

## Supporting information



Supporting Information

## Data Availability

The data that support the findings of this study are available from the corresponding author upon reasonable request.
