# Supplementary material for: Reversible Carrier Modulation in InP Nanolasers by Ionic Liquid Gating with Low Energy Consumption
Source: Adv Sci (Weinh). 2024 Dec 16;12(8):2412340. doi: 10.1002/advs.202412340 (PMC11848537; doi:10.1002/advs.202412340)
Supplement: Supplementary file 1 — Supporting Information [file ADVS-12-2412340-s001.docx]

**Supporting Information**

**Reversible Carrier Modulation in InP Nanolasers by Ionic Liquid Gating with Low Energy Consumption**

Chia-Hung Wu^1,2^, Chi-Wen Chen^3^, Hung-Jung Shen^4^, Hsiang-Yu Chuang^4^, Hark Hoe Tan^5^, Chennupati Jagadish^5^, Tien-Chang Lu^6^, Satoshi Ishii^2^, and Kuo-Ping Chen^4,*^

1 College of Photonics, National Yang Ming Chiao Tung University, 301 Gaofa 3rd Road, Tainan 71150, Taiwan

2 International Center for Materials Nanoarchitectonics (MANA), National Institute for Materials Science (NIMS), 1-1 Namiki, Tsukuba, Ibaraki 305-0044, Japan

3 Institute of Photonic System, College of Photonics, National Yang Ming Chiao Tung University, 301 Gaofa 3rd Road, Tainan 71150, Taiwan

4 Institute of Photonics Technologies, National Tsing Hua University, Hsinchu 300, Taiwan

^5^ ARC Centre of Excellence for Transformative Meta-Optical Systems, Department of Electronic Materials Engineering, Research School of Physics, The Australian National University, Canberra, ACT, 2600, Australia

^6^ Department of Photonics, College of Electrical and Computer Engineering, National Yang Ming Chiao Tung University, Hsinchu 30010, Taiwan

*kpchen@ee.nthu.edu.tw

KEYWORDS: InP nanowire laser, Ionic liquid, Carrier modulation, Flexible substrate

**Fabrication:**


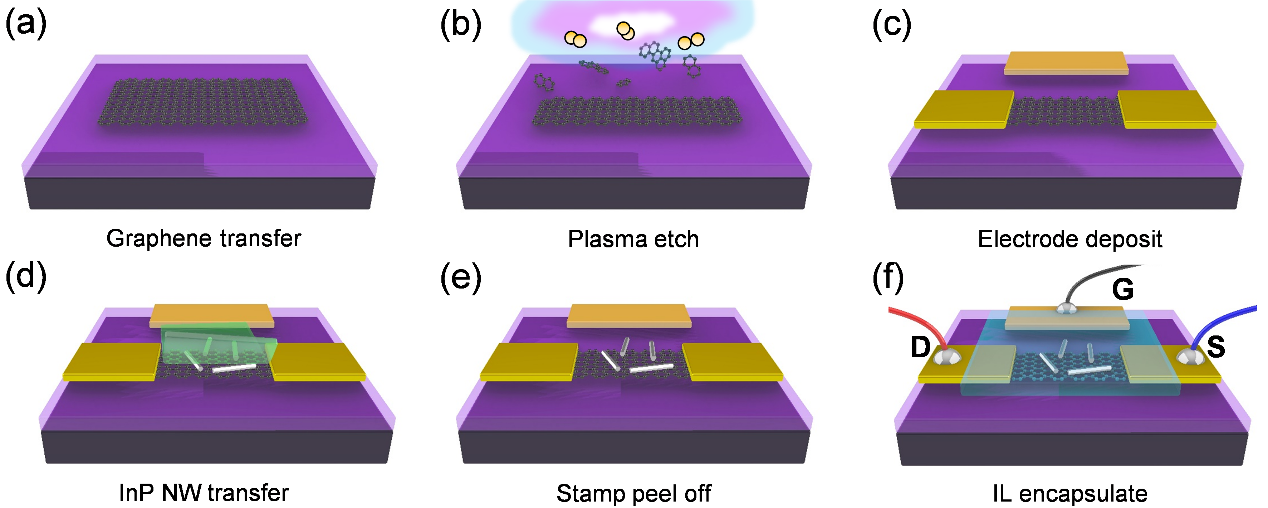


**Figure S1.** Schematic of Device A fabrication steps. (a) Wet-transfered graphene on a substrate then patterned with photoresist as a protective mask. (b) Oxygen plasma etch to form graphene channel and removal of photoresist mask with acetone. (c) Thermal deposition of electrodes with a metal mask. (d) Transfer of MOCVD grown InP NWs with PDMS stamp onto graphene channel. (e) Peeling off PDMS stamp very slowly to avoid damage of graphene channel. (f) Drop casting 1 μL IL and encapsulate with a cover glass.

**Home made transfer platform:**


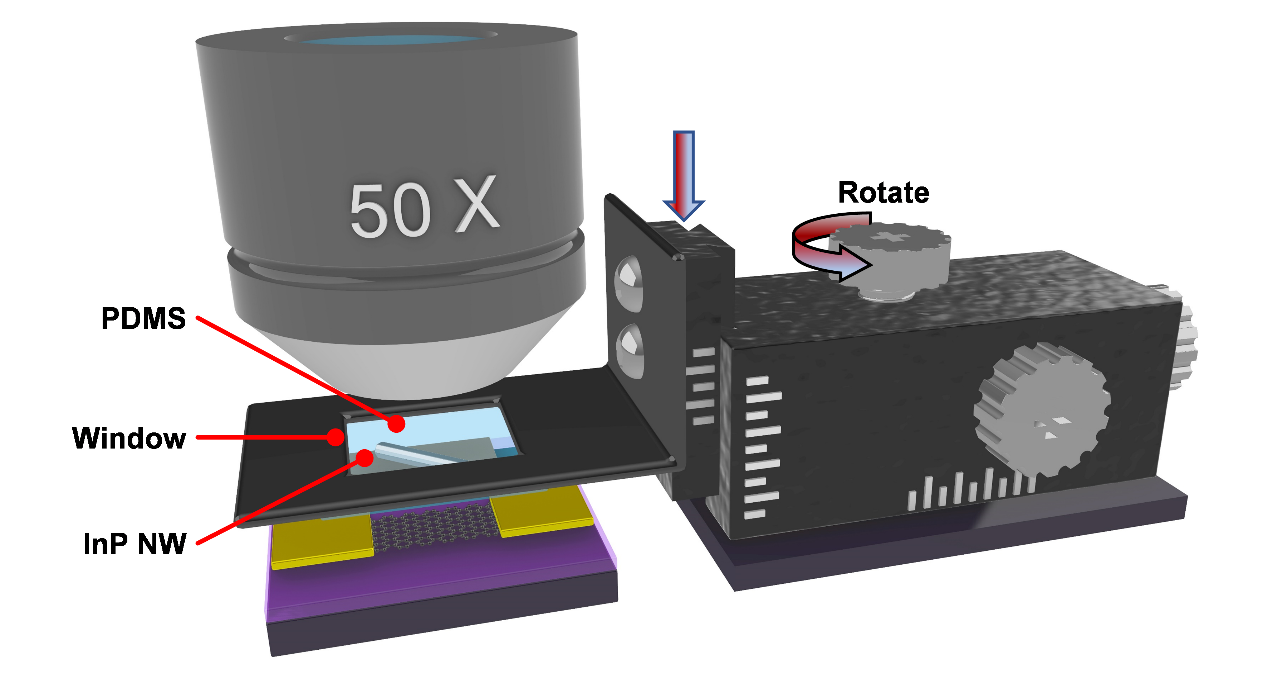


**Figure S2.** Schematic of home made transfer platform. An L shaped stage with a window was fixed to a probe station to enable precise transfer. The PDMS stamp with InP NWs was attached to the window of the stage and manipulated through a 50× objective lens and a camera. When the PDMS stamp was in contact with the sample, the stamp was dettached from the L shaped stage and stayed on the sample due to larger contact area. The stamp was then removed by peeling off slowly to leave the InP nanowires on the designated area.


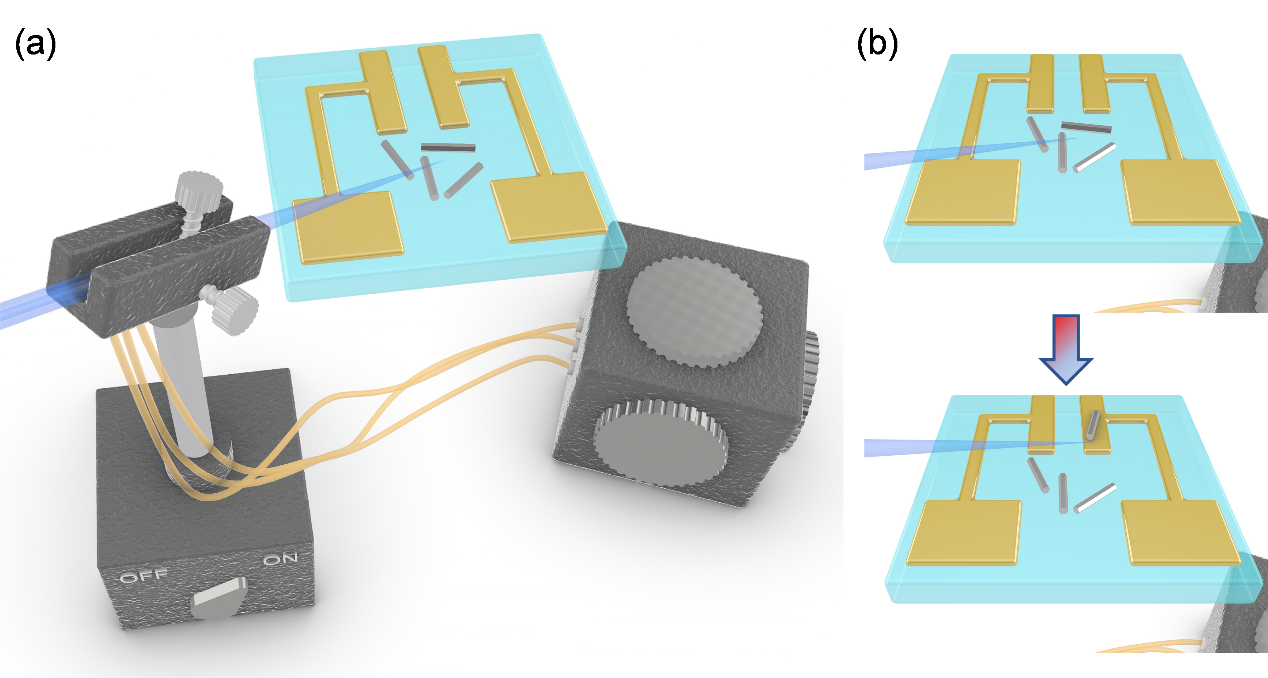


**Figure S3.** Schematic of micro manipulation of InP NWs. (a) Glass probe mounted on an 1 μm accuracy three-axis oil hydraulic micromanipulator (MMO-203, Narishige Scientific Instrument Lab., Japan). (b) InP NW was pushed onto the electrode with the micromanipulator.

**Measurements:**


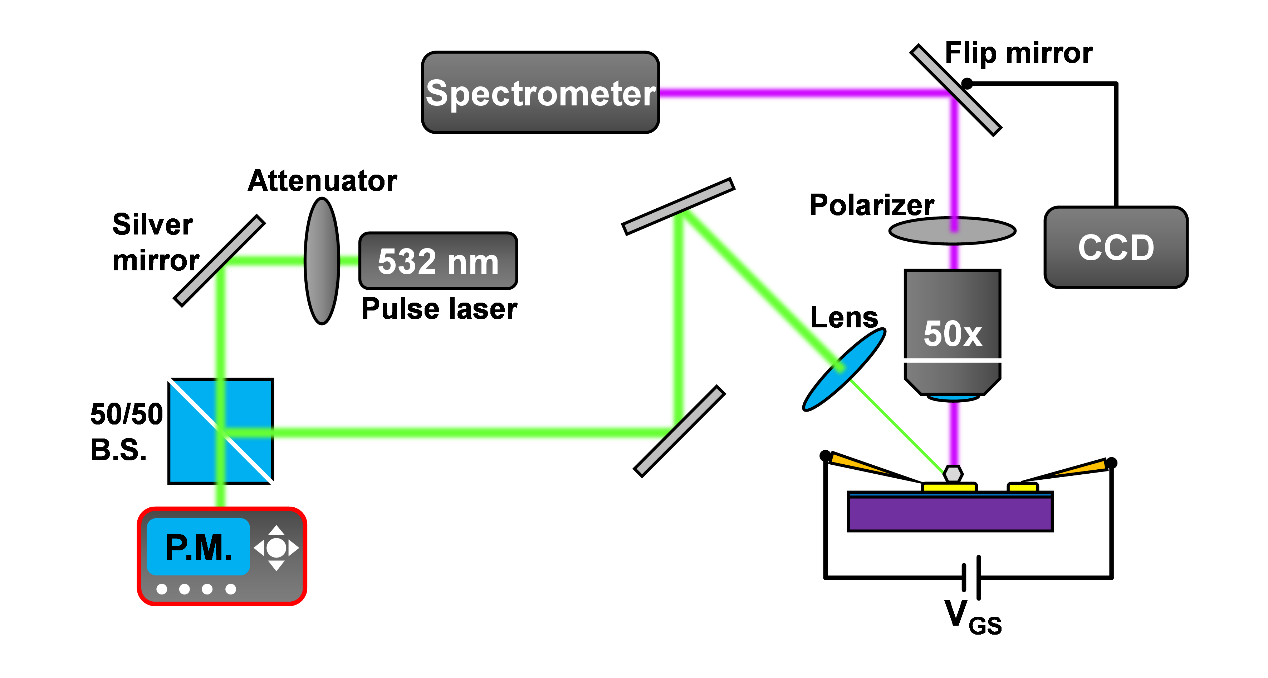


**Figure S4.** Schematic of measurement and gating setup. 532 nm pulse laser power was reduced with a broad band disk attenuator and monitored with a power meter. The reflected laser beam from the 50/50 beam splitter was guided by an adjustable silver mirrors and focused by a 5 cm focal length convex lens onto the sample. Emission signals form the InP nanowire was collected through an 50× objective then into the spectrometer. Polarizer in the figure was only applied for Fig. 1 (e) inset. Gate voltage was applied with a source meter (B2901A).

**Band diagram:**

In this section, we discuss briefly about how the threshold and wavelength of InP NW lasing were modulated when subjected to IL gating. A donor band was formed in vicinity of the conduction band at n-type doping. At room temperature (300 K), a fraction of electrons from the donor band filled up the lower enrgy states of the conduction band. This resulted in a slight upshift in the effective metastable state. After the system was excited by a 532 nm pulse laser, the interband transition of electrons from the valence band took place, filling the conduction band to a higher energy state as lower energy states were occupied. Once these excited carriers relax, some fell back to the ground state producing photoluminescence and some fell into the metastable state. With the increase of pumping energy, accumulated electrons in the metastable state led to population inversion and stimulated emission. Compared to intrinsic NWs, n-doped NW had additional electrons readily occupied at higher energy states when subjected to pumping. Therefore, the threshold for stimulated emission was far less than undoped and p-doped cases. Additionally, as previously mentioned, lower energy states in the conduction band was occupied due to doping. The photo-excited carriers was then filled up at even higher energy states, causing a higher energy of radiation loss when the carriers recombined at ground state. This phenomenon explains the blue shift observed in the results measured in our experiment and refers to the Moss-Burstein effect. For the p-doped InP NWs, an acceptor band was formed in vicinity of valence band, causing the effective bandgap to decrease. Once the system was illuminated, the excited carriers from the conduction band was recombined around the acceptor and valence band causing the red shift in emission spectrums. Additional acceptors in the sytem caused the threshold of lasing to increase as photo-excited carriers may have recombined before the interband transition.


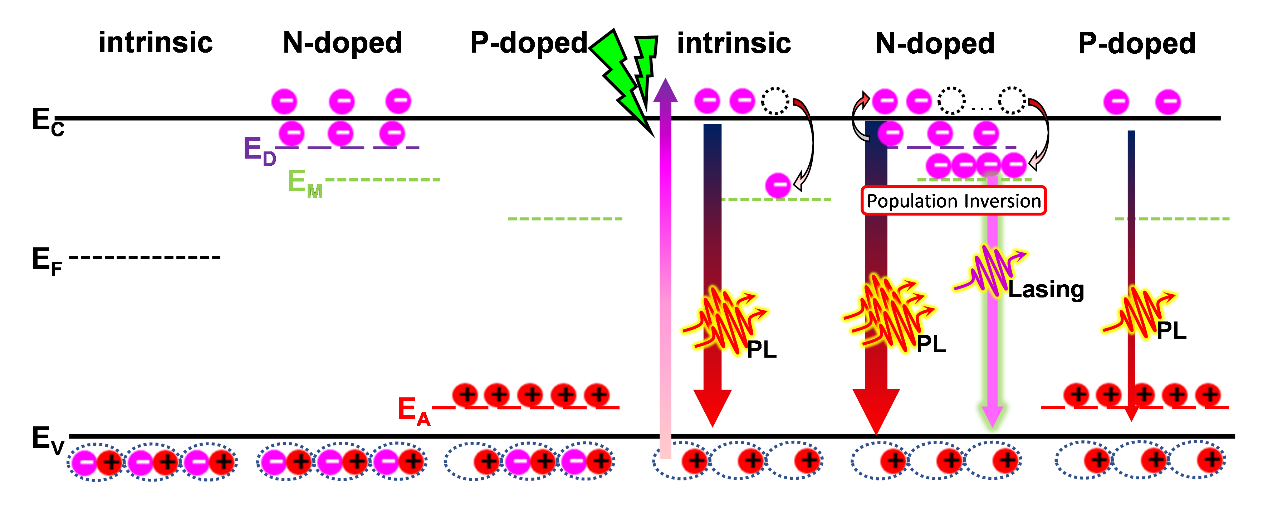


**Figure S5.** Visualized band diagram when InP nanowire subjected to doping. On the right half of the figure seperated by a green lightning icon refers to after illumination of laser. Upward arrows refer to the interband transition of excited electrons. Downward arrows refer to the relaxation and recombination of electrons. Abbreviations are listed as follows. Conduction bamd (E_C_), valence band (E_V_), Fermi level (E_F_), metastable state (E_M_), donor band (E_D_), acceptor band (E_A_) and photoluminescence (PL).

**Finite Difference Eigenmode Solver – mode analysis:**

The emmisive mode investigation in the NW cavity was obtained through the Finite Difference Eigenmode Solver (FDE). We calculated the group index $n_{g}=5.24$ with the following equation from experimental datas.

$$n_{g}=\frac{{\lambda_{lasing}}^{2}}{2*\Delta\lambda*L_{NW}}$$

Where $n_{g}$ is the group index, $\lambda_{lasing}$ is the lasing wavelength (881.48 nm), $\Delta\lambda$ is the mode spacing (5.7 nm) and $L_{NW}$ is the NW length (13 μm). After the group index is extracted from experimental data, FDE simulations is conducted as shown in **Figure S6** (a). Modal analysis shows different guided modes propagating in the NW as shown in **Figure S7**. By comparing the experimental and simulated group index as shown in **Table S1**, the mode propagating in the NW under V_GS_ = 3.5 V is HE31_b_.

**
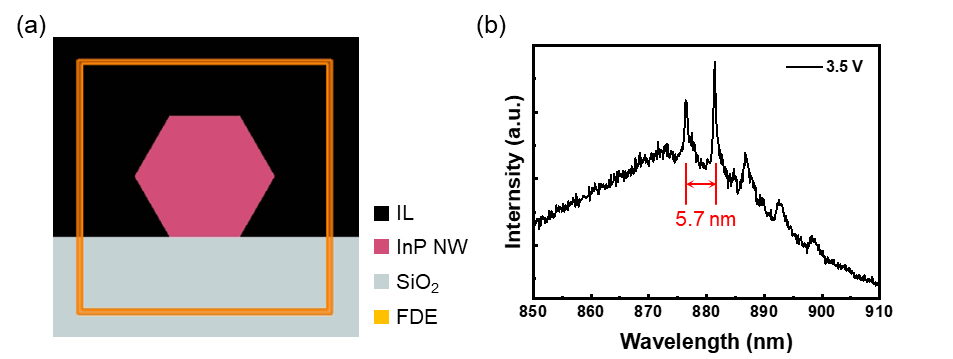
**

**Figure S6.** (a)Finite Difference Eigenmode Solver (FDE) of InP NW in Device A. (b) Emission spectrum of Device A just above threshold when immersed in IL. The lasing peak is at 881.48 nm and the mode spacing is 5.7 nm.


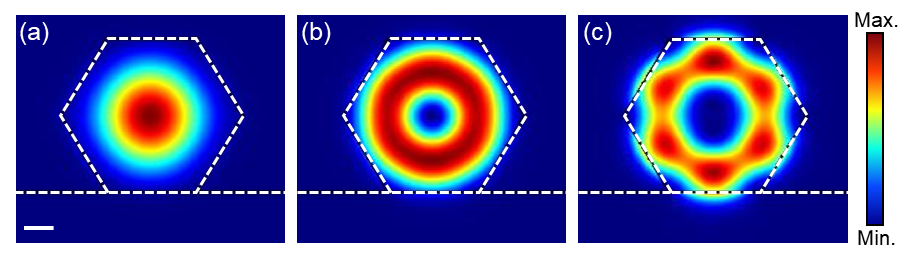


**Figure S7.** Finite Difference Eigenmode Solver (FDE) simulation electric field intensity profiles of guided modes in InP NW immersed in IL under V_GS_ = 3.5 V. (a) HE11_a_ (b) TE01 (c) HE31_b_. The scale bar is 100 nm.

|  | Mode HE11_a_ | Mode TE01 | Mode HE31_b_ | Experimental |
| --- | --- | --- | --- | --- |
| Group index (n_g_) | 4.114 | 4.289 | 5.241 | 5.243 |
| Effective index (n_eff_) | 3.263 | 3.106 | 2.569 | NA |

**Table S1.** Group index (n_g_) and effective refractive index (n_eff_) of different modes under V_GS_ = 3.5 V.

**Gated InP Nanowire Refractive Index Approximation:**

To calculate the refractive index variation in the nanowire cavity under different gating voltages, we first obtain the mode number m by the following equation. Derived from the resonance condition in a waveguide or a resonator.

$$m\lambda=2n_{eff}L_{NW}$$

Next, we estimate the effective refrative index variation with the lasing wavelength shift under mode HE31b.

$$m(\lambda_{3.5V}-\lambda_{0V})=2{(\Delta n}_{eff})L_{NW}$$

We can see that the relative change in resonant wavelength is proportional to the relative change in refractive index. Note that this relationship is applicable if all other parameters (such as cavity length and mode number) remain constant.

By applying the abovementioned equation, the lasing wavelength was extracted from V_GS_ = 3.5 V and 0 V as 881.477 nm and 882.143 nm, respectively. The refractive index of the IL layer was kept constant at $n_{IL}=1.424$ due to negligible changes as discussed in previous sections. The HE31_b_ mode as depicted in **Figure S7** (c) effective refractive index change $\Delta n_{eff-InP}(3.5 V)$ was estimated to be $-2\times{10}^{-3}$. Given the minimal estimated refractive index change in the ionic liquid layer surrounding the nanowire, we attribute the observed refractive index variation primarily to changes in carrier concentration within the nanowire.

**Gated InP Nanowire Carrier Concentration Approximation:**

To estimate the carrier concentration change $\Delta N$, the following equation derived from Drude model was employed.[1]

$$\Delta n_{eff}=\frac{{-e}^{2}\lambda_{0}^{2}}{8\pi^{2}c^{2}\epsilon_{0}n_{r}}(\frac{\Delta N_{e}}{m_{ce}^{*}}+\frac{\Delta N_{h}}{m_{ch}^{*}})$$

Where $\epsilon_{0}:8.85$‧10^-12^ F/m, e: 1.60‧10^-19^ C, m_0_: 9.11‧10^-31^ kg, $m_{cⅇ}^{*}:0.08m_{0}$, $m_{ch}^{*}:0.6m_{0}$, λ_0_: 882.14 nm and n_r_: 2.569

Due to N-type doping at V_GS_ = 3.5 V, the variation of positive carriers could be neglected, giving us $\Delta N_{e}(3.5 V)=1.18x{10}^{18} {cm}^{-3}$.

**Biased Ionic Liquid Refractive Index Approximation:**

The refractive index change of ionic liquids under bias is indeed present, but the variation in value is small and negligible. To estimate the refractive index change of biased ionic liquids,[2-4] we need to find the relation between the refractive index and the ionic molecular concentration. Hence the following equation was employed.

$$\Delta n=k\times\Delta N$$

Where $\Delta n$ is the refractive index change, k is the refractive index sensitivity constant and $\Delta N$ is the carrier concentration change in the liquid. Given that concentration changes are more significant in the EDL layer, we assume that all variations stem from this layer rather than the bulk solvent. Here, we approximate the change in liquid ion concentration change equal to the InP carrirer concentration change when biased at 3.5 V, that is $1.18\times{10}^{18}{cm}^{-3}$.

To derive k, we employ the Lorentz- Lorenz equation which relates the refractive index and density of a dielectric.[5]

$$\frac{n^{2}-1}{n^{2}+2}=\frac{4\pi}{3}N\alpha$$

Where $\alpha$ is the average molecular polarizability of the particles in the solvent.[6, 7] We first take differentiate with respect to N.

$$\frac{d}{dN}\left( \frac{n^{2}-1}{n^{2}+2} \right)=\frac{4\pi}{3}\alpha$$

By applying the Quotient and chain rule, we could obtain the following equation.

$$\frac{dn}{dN}=k=\frac{2\pi\alpha{(n^{2}+2)}^{2}}{9n}$$

Substitute back to the first equation, we could obtain the following equation.

$$\Delta n=\frac{2\pi\alpha{(n^{2}+2)}^{2}}{9n}\times\Delta N$$

The estimated refractive index change in the EDL layer for the ionic liquid is approximtely $1\times{10}^{-4}$.

**Long-term Device Stability:**

For long-term device stability, our proposed IL-gated nanowire system withstands at least 30 ON-OFF (V_GS_ = +2.2 V and 0 V) cycles, as shown in the figure. The pumping laser power was kept consistent and continuously on throughout the experiment. In the first three measurements, we observed an climbing in lasing intensity, indicating carrier accumulation around electrodes. Each duration of ON-OFF cycles were approximately 5 seconds. The system withstands 30 cycles and we see a slow decay in lasing intensity which we believe is caused by modal conversion introduced by excessive carrier accumulation inside the nanowire cavity. To refresh the system, we tried turning off the pumping source and rested the system for 5 minutes. The InP nanowire was back to its initial state, which we believe is due to induced carrier relaxation and diffusion. Thus, alternating positive and negative bias pulses should help control the carrier type in the nanowire cavity, resulting in more stable ON-OFF states. Additionally, we believe that by encapsulating the ionic liquid, the system’s stability could be improved by preventing exposure to atmospheric moisture, which has a strong effect on the ionic liquid’s chemical stability. For data homogeneity, we investigated up to 15 nanowires on multiple substrates, all of which demonstrated modulation capability under the proposed concept. However, the required modulation bias varied due to the intrinsic threshold of each nanowire, which depends greatly on its geometry. This variation could be minimized by carefully controlling the growth process to produce more uniform nanowires.

**Figure S8.** Repetitive ON-OFF cycles of the proposed device.

**Recombination Rates of Gated InP nanolaser:**

To estimate the recombination rate of the carriers in the excited state, we have conducted the time resolved photoluminescence (TRPL) experiment on three gating voltages: V_GS_ = 2.4 V, 0 V and -1 V. When the InP nanowire is N-type doped (V_GS_ = 2.4 V), the increased carrier concentration leads to saturation of traps responsible for heat or phonon generation, which is known as non-radiative recombination. By filling up these traps, we could expect an increase in internal quantum efficiency and an increase in carrier lifetime as more carriers in the excited state could channel through the radiative recombination. On the contrary, under P-type doping scenarios, the hole concentration is increased, decreasing electron concentration. This leads to early recombination of carriers through the non-radiative channel and shortens carrier lifetime. From the measurement results, lifetime of carriers are 0.43 ns, 0.35 ns and 0.25 ns under V_GS_ = 2.4 V, 0V and -1 V respectively. This result could be explained by the Schockley-Read-Hall (SRH) recombination model.[8] The lifetime of carriers $\tau$ could be obtained from the rates of radiative ${1/\tau}_{r}$ and non-radiative $1/\tau_{nr}$ pathways.

$$\frac{1}{\tau}=\frac{1}{\tau_{r}}+\frac{1}{\tau_{nr}}$$

The non-radiative recombination rate is defined as:

$$R_{nr}=\frac{n}{\tau_{trap}}$$

Where $\tau_{trap}$ is the trap-assisted recombination lifetime, often shorter at lower carrier densities. At higher carrier densities, the traps are filled, leading to saturation of non-radiative recombination rate $R_{nr}$.

The radiative recombination rate is defined as:

$$R_{r}=Bn^{2}$$

Where *B* is the radiative recombination coefficient. As the carrier concentration *n* increases, $R_{r}$ overrates $R_{nr}$, which increases the radiative contribution and extending the effective fluorescence lifetime.

The TRPL experiment excitation laser was a 405 nm ps laser (LDH-D-C-405, Picoquant), the emitted signal was filtered with a 580 nm long pass filter and collected with avalanche photo diode (MPD PDM Series). The APD output is connected to a timing module (PicoHarp 150, PicoQuant), which records the arrival time of each photon.

**Figure S9.** Time resolved photoluminescence (TRPL) of the proposed device under different gating voltages.

**References:**

[1] S.-L. Wang, S. Wang, X.-K. Man, and R.-M. Ma, "Loss and gain in a plasmonic nanolaser," *Nanophotonics,* vol. 9, no. 10, pp. 3403-3408, 2020.

[2] A. Okada, T. Kobayashi, and E. Tokunaga, "Interfacial Pockels Effect of Solvents with a Larger Static Dielectric Constant than Water and an Ionic Liquid on the Surface of a Transparent Oxide Electrode," *Applied Sciences,* vol. 12, no. 5, p. 2454, 2022.

[3] Y. Nosaka, M. Hirabayashi, T. Kobayashi, and E. Tokunaga, "Gigantic optical Pockels effect in water within the electric double layer at the electrode-solution interface," *Physical Review B—Condensed Matter and Materials Physics,* vol. 77, no. 24, p. 241401, 2008.

[4] I. Datta *et al.*, "Low-loss composite photonic platform based on 2D semiconductor monolayers," *Nature Photonics,* vol. 14, no. 4, pp. 256-262, 2020.

[5] T. G. Mayerhöfer, A. Dabrowska, A. Schwaighofer, B. Lendl, and J. Popp, "Beyond Beer's law: why the index of refraction depends (almost) linearly on concentration," *ChemPhysChem,* vol. 21, no. 8, pp. 707-711, 2020.

[6] P. Kubisiak, P. Wróbel, and A. Eilmes, "How temperature, pressure, and salt concentration affect correlations in LiTFSI/EMIM-TFSI electrolytes: a molecular dynamics study," *The Journal of Physical Chemistry B,* vol. 125, no. 44, pp. 12292-12302, 2021.

[7] V. Lesch, Z. Li, D. Bedrov, O. Borodin, and A. Heuer, "The influence of cations on lithium ion coordination and transport in ionic liquid electrolytes: a MD simulation study," *Physical Chemistry Chemical Physics,* vol. 18, no. 1, pp. 382-392, 2016.

[8] W. Shockley and W. Read Jr, "Statistics of the recombinations of holes and electrons," *Physical review,* vol. 87, no. 5, p. 835, 1952.
